# Structural causes of pattern formation and loss through model-independent bifurcation analysis

**DOI:** 10.1007/s00285-025-02296-9

**Published:** 2025-10-27

**Authors:** Liam D. O’Brien, Adriana T. Dawes

**Affiliations:** 1 Department of Mathematics, The Ohio State University, 231 W 18th Ave, Columbus, 43210 Ohio USA; 2 Department of Molecular Genetics, The Ohio State University, 484 W 12th Ave, Columbus, 43201 Ohio USA

**Keywords:** Bifurcation theory, Network dynamics, Pattern formation, Developmental biology, Signaling networks, 34A34, 24C14, 34C23, 92C15, 92C40, 92C42

## Abstract

During development, precise cellular patterning is essential for the formation of functional tissues and organs. These patterns arise from conserved signaling networks that regulate communication both within and between cells. Here, we develop and present a model-independent ordinary differential equation (ODE) framework for analyzing pattern formation in a homogeneous cell array. In contrast to traditional approaches that focus on specific equations, our method relies solely on general assumptions about global intercellular communication (between cells) and qualitative properties of local intracellular biochemical signaling (within cells). Prior work has shown that global intercellular communication networks alone determine the possible emergent patterns in a generic system. We build on these results by demonstrating that additional constraints on the local intracellular signaling network lead to a single stable pattern which depends on the qualitative features of the network. Our framework enables the prediction of cell fate patterns with minimal modeling assumptions, and provides a powerful tool for inferring unknown interactions within signaling networks by analyzing tissue-level patterns.

## Introduction

Pattern formation is a hallmark of developmental biology, where cells within a tissue or organism differentiate in a precise spatial arrangement to form complex structures. This process is essential for cell fate specification, tissue development, and morphogenesis; and understanding the molecular causes of pattern formation has promising applications in regenerative medicine, tissue engineering, and the treatment of congenital malformations (Chuong and Richardson 2009).

During development, a group of cells with the same developmental potential will receive a signal called a *morphogen* that prompts them to communicate and form a pattern of cell types. These local patterning events occur sequentially, with each iteration creating progressively finer cellular patterns across the developing organism. Recent ordinary differential equation (ODE) models have considered one iteration, focusing on how changes in cell-communication and chemical kinetics affect the cellular pattern (Williamson et al. 2021; O’Dea and King 2012; de Back et al. 2013; Fisher et al. 2005; Irons et al. 2010; Giurumescu et al. 2006; Vasilopoulos and Painter 2016; Hadjivasiliou et al. 2016). Most models investigate the Notch signaling pathway, which is a key signaling pathway involved in contact-mediated cell-communication. Although the full complexity of the Notch pathway is difficult to model, simplified systems – such as the model from Collier et al. (1996) – have offered valuable insights into how cell-communication can give rise to fine-grained patterns.

Over time, modeling changes have produced further understanding. For instance, O’Dea and King (2012) incorporated morphogens that act as bifurcation parameters, showing how an initially homogeneous steady state can be destabilized, leading to the formation of patterns. They also derived a continuum model to account for morphogen gradients, showing how tissues with different chemical gradients form different patterns. Williamson et al. (2021) used a similar approach and demonstrated that the structure of communication networks among cells determines the types of fine-grained patterns that can emerge. Others have shown via simulations that coarse-grained patterns such as spots and stripes can arise if there is long-range signaling in the Notch pathway (Vasilopoulos and Painter 2016; Hadjivasiliou et al. 2016).

Despite the usefulness of these models, they all rely on specific equations; therefore, there are possible dynamics that the models cannot represent due to potentially incomplete assumptions about the system, including assumptions about reaction rates and the molecular pathways involved (e.g. focusing on Notch signaling). Moreover, although model investigation has helped with understanding which parameters are important for pattern formation, the inherent complexity of any model prohibits us from determining which parameters are necessary or sufficient for a pattern. Recent advances in network theory (Golubitsky and Stewart 2023) allow a more thorough investigation of pattern formation in many cell-communication networks. By ignoring all unnecessary information and focusing on the connectivity of cells (i.e. which cells influence each other), one can predict all possible patterns of cell fates that can emerge in a generic system (Wang and Golubitsky 2004). Additionally, we provide simple conditions – related to qualitative features of the chemical signaling network – that are both necessary and sufficient for a pattern of cell fates to form under our assumptions.

Using our theory, we find that despite the limited chemical signaling pathways involved in development, cells can generate diverse patterns by reorganizing themselves, thus validating and extending the work of Williamson et al. (2021), Vasilopoulos and Painter (2016), and Hadjivasiliou et al. (2016). On the other hand, cells whose organization is constrained can form various patterns by using different chemical signaling mechanisms, as suggested in de Back et al. (2013). We identify conditions under which cells will fail to form stable patterns, instead remaining in a homogeneous steady state, oscillating synchronously, or forming oscillating patterns. Importantly, our theory shows that the chemical kinetics proposed in the Collier model are not the only kinetics that can lead to pattern formation. Finally, we are able to infer possible biochemical interactions from an observed pattern, which can reveal previously unknown interactions in biochemical signaling pathways.

The paper is organized as follows. In Sect. 2, we state our biological assumptions and introduce our mathematical framework, including the theory of network dynamics. We highlight how bifurcations in networks can give rise to patterns corresponding to eigenvectors of the system. In Sect. 3, we show that qualitative features of chemical signaling determine the stability of the initially homogeneous steady state and dictate the critical eigenvectors that lead to pattern formation. In Sect. 3.2, we apply our theory to various biological contexts and predict patterns using minimal modeling assumptions. We show how the same chemical signaling pathway can produce different patterns depending on the cell-communication network. Finally, in Sect. 3.3, we demonstrate how our framework can be used to infer properties of biochemical signaling pathways from observed patterns in tissues.

## Mathematical methods

### General model assumptions

We make the following general assumptions to guide the construction of our network models and their associated internal dynamics.

1. The cells are well-mixed compartments that can be characterized by the concentration of multiple chemical species inside the cell.
2. The concentrations of chemical species within the cells change smoothly over time.
3. The cells begin in a nearly identical state.
4. The external signals to each cell are identical.
5. The cells are in a sufficiently uniform lattice structure. (The lattice may be in 1,2, or 3 spatial dimensions).
6. The cells average signals from their neighbors.

Assumptions (1) and (2) allow us to represent our system of cells with ordinary differential equations. Assumptions (3)-(6) allow us to represent our system with a *strongly connected, regular network* as defined in Sect. 2.

### Network definitions and basic properties

Our approach emphasizes the role of network structure in pattern formation, so we outline key definitions and properties that will be used throughout the paper (for a fully rigorous treatment, see Nijholt et al. (2016); Golubitsky and Stewart (2023)).

#### Definition 1

*(Regular Networks)* A regular network is a finite directed graph $\mathcal {N}=(\mathscr {V},\mathscr {A},s,t)$ where

1. $\mathscr {V}$ is the set of nodes
2. $\mathscr {A}$ is the set of arrows
3. $s: \mathscr {A} \rightarrow \mathscr {V}$ is the source map
4. $t:\mathscr {A} \rightarrow \mathscr {V}$ is the target map.

Additionally, the number of input arrows to each node is the same (i.e. $|t^{-1}(\{v_1\})|=|t^{-1}(\{v_2\})|$ for all $v_1,v_2\in \mathscr {V}$). We call the number of input arrows the valence of the network.

**Fig. 1:**
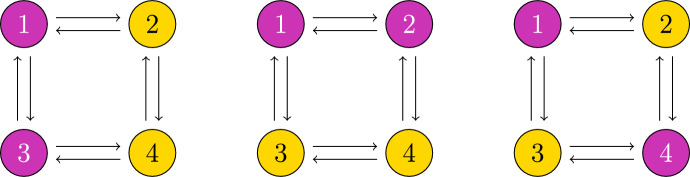
Each of these diagrams represents a balanced coloring on the regular four node network that is depicted. Indeed, in the left and center images, each yellow (light) node gets one input from pink (dark) and one input from yellow, while both pink nodes get one input from pink and one from yellow. On the right, each pink node gets two inputs from yellow, and each yellow node gets two inputs from pink

For example, each network shown in Fig. 1 (ignoring colors) is a regular network because every node has two input arrows.

There is a class of ODEs called *admissible ODEs* that is naturally associated with a regular network $\mathcal {N}$. Suppose that each node $v \in \mathscr {V}$ has an associated “state” $\textbf{x}_v \in \mathbb {R}^s$ – where $\mathbb {R}^s$ is called the *node space*. Then the state of an *n*-node network with node space $\mathbb {R}^s$ can be described by $\textbf{x}_{\mathcal {N}} =(\textbf{x}_v)_{v\in \mathscr {V}} \in \mathbb {R}^{ns}$.

Next, suppose that each node *v* is only influenced by itself and nodes *w* such that there is an arrow from *w* to *v* (i.e. $s(a)=w$ and $t(a)=v$ for some $a\in \mathscr {A}$). Let $\{v_j^i\}_{1\le j \le \nu }$ denote the set of input nodes to $v_i$ (i.e. nodes satisfying $s(a)=v_j^i$ and $t(a)=v_i$ for some $a\in \mathscr {A}$).

#### Definition 2

*(Admissible ODE for a Regular Network)* An ODE is called admissible for the regular network $\mathcal {N}$ if for some smooth function *f*, the dynamics of each node can be written in the form

$$\begin{aligned} \dot{\textbf{x}}_{v_i} = f(\textbf{x}_{v_i}, \overline{\textbf{x}_{v_1^i},...,\textbf{x}_{v_{\nu }^i}}) \end{aligned}$$

where the overline indicates that *f* is symmetric in the arguments 2 through $\nu +1$.

#### Remark 1

To model a system with a regular network, each node needs to behave identically, and they need to communicate in an identical manner. In the network, this is represented by the identical node and arrow types. See Fig. 1 (ignoring colors).

#### Definition 3

*(Balanced Colorings of Regular Networks)* For a regular network $\mathcal {N} = (\mathscr {V},\mathscr {A},s,t)$, assign every vertex a color $\{1,2,...,k\}$ via the coloring map

$$\begin{aligned} \kappa : \mathscr {V} \rightarrow \{1,2,...,k\}. \end{aligned}$$

This coloring is balanced if for every $v_1,v_2\in \mathscr {V}$ with $\kappa (v_1)=\kappa (v_2)$ there exists a color-preserving bijection between their input nodes:

$$\begin{aligned} \alpha : \{s(a)\}_{t(a)=v_1} \rightarrow \{s(a)\}_{t(a)=v_2}. \end{aligned}$$

Intuitively, a coloring is balanced if for any two nodes $v_1,v_2$ with the same color, the set of nodes influencing $v_1$ has exactly the same colors, with the same frequency, as the set of nodes influencing $v_2$. See Fig. 1 for examples of balanced colorings.

#### Definition 4

*(Polysynchrony Subspace)* Let $\bowtie$ (pronounced “bowtie”) be a balanced coloring of a regular network $\mathcal {N} = (\mathscr {V}, \mathscr {A}, s,t)$ given by the coloring map $\kappa$. The associated polysynchrony subspace is the set

$$\begin{aligned} \Delta _{\bowtie } = \{\textbf{x}_{\mathcal {N}}: \textbf{x}_{v_1} = \textbf{x}_{v_2} \ \text {whenever} \ \kappa (v_1) = \kappa (v_2)\}. \end{aligned}$$

**Fig. 2:**
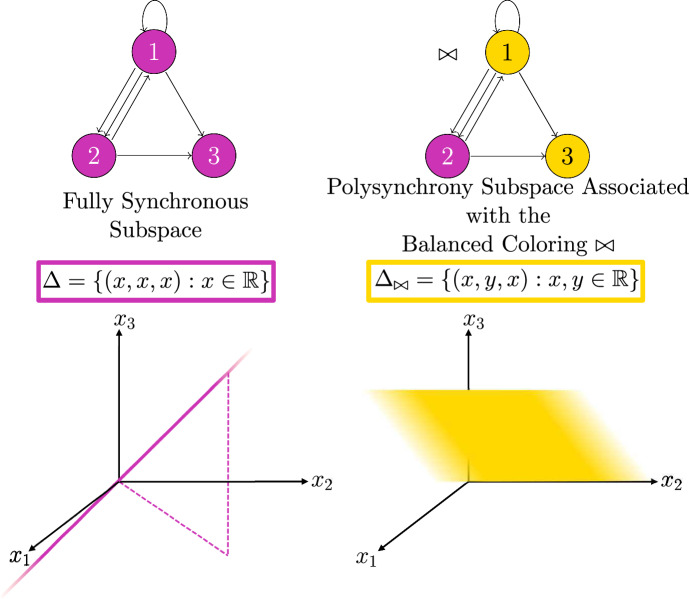
Classically, ODEs are imagined in phase space. Considering variables from a network perspective allows us to consider synchronous nodes, while maintaining a natural correspondence to phase space. If the node space is $\mathbb {R}$, then the phase space of any admissible ODE for the depicted network is $\mathbb {R}^3$. Balanced colorings represent patterns of synchrony that correspond to polysynchrony subspaces of the phase space. For example, if all cells are equal for all time, the trajectory of a solution to the ODE is inside the fully synchronous subspace $\Delta$ depicted on the bottom left. If $x_1(t) = x_3(t)$ for all time, we can represent the pattern of synchrony with the balanced coloring $\bowtie$ in the network (top right); and the coloring corresponds to the polysynchrony subspace $\Delta _{\bowtie }$ depicted on the bottom right

See Figs. 1, 2 for examples of balanced coloring and corresponding polysynchrony subspaces.

#### Definition 5

*(Flow Invariance)* Let $F: X \rightarrow X$ be a smooth vector field. A subspace $E \subset X$ is flow-invariant under *F* if $F(E) \subset E$.

#### Proposition 1

(Polysynchrony Subspaces are Flow-Invariant) Let $\dot{\textbf{x}}_{\mathcal {N}} = F(\textbf{x}_{\mathcal {N}})$ be an admissible ODE for the network $\mathcal {N}$. Every polysynchrony subspace given by a balanced coloring is flow-invariant under *F*.

#### Proof

See Golubitsky and Stewart (2023), Theorem 8.16. $\square$

#### Remark 2

Even when a network is highly symmetric, there can be flow-invariant subspaces given by balanced colorings, which are not fixed-point subspaces of a symmetry subgroup. Therefore, network theory captures different structure than equivariant theory, making it more appropriate for analyzing cell-communication networks (Golubitsky and Stewart (2023), Ch. 16).

The balanced colorings in Fig. 1 from left to right correspond to the polysynchrony subspaces

$$\begin{aligned} \Delta _1&= \{(\textbf{x},\textbf{y},\textbf{x},\textbf{y})\in \mathbb {R}^{4s}: \ \textbf{x},\textbf{y}\in \mathbb {R}^s\} \\ \Delta _2&= \{ (\textbf{x},\textbf{x},\textbf{y},\textbf{y})\in \mathbb {R}^{4s}: \ \textbf{x},\textbf{y}\in \mathbb {R}^s\} \\ \Delta _3&= \{(\textbf{x},\textbf{y},\textbf{y},\textbf{x})\in \mathbb {R}^{4s}: \ \textbf{x},\textbf{y}\in \mathbb {R}^s\}. \end{aligned}$$

For regular networks, coloring every node the same is always balanced, and the associated polysynchrony subspace is called the *fully synchronous subspace*, which we denote $\Delta$. If there is an equilibrium $\textbf{x}_{\mathcal {N}}$ of an admissible ODE satisfying $\textbf{x}_{\mathcal {N}}\in \Delta$, we say that the equilibrium is *fully synchronous*.

### Eigenvalues depend on cell-level dynamics and network structure

We show that at a synchronous steady state, patterned solutions can only emerge through a bifurcation. Because bifurcations require at least one eigenvalue with zero real part – and the corresponding eigenvectors provide qualitative information about emerging solutions – we also present a method for computing eigenpairs of admissible ODEs on regular networks.

Suppose $\dot{\textbf{x}}=F(\textbf{x},\lambda )$ is an admissible ODE for a regular network, where $\lambda \in \mathbb {R}$ is a bifurcation parameter. Let $(\textbf{x}^*,\lambda ^*)\in \Delta \times \mathbb {R}$ be a synchronous equilibrium of the system satisfying $F(\textbf{x}^*,\lambda ^*)=0$. If the Jacobian $\textbf{J}=(dF)_{(\textbf{x}^*,\lambda ^*)}$ is nonsingular (e.g. at a stable equilibrium), the implicit function theorem implies that there is a unique branch of solutions $(\textbf{x}(\lambda ),\lambda )$ satisfying $F(\textbf{x}(\lambda ),\lambda )=0$ in a neighborhood of $(\textbf{x}^*,\lambda ^*)$. Furthermore, if $\textbf{J}\big |_{\Delta }$ is nonsingular then the unique solution $(\textbf{x}(\lambda ),\lambda )$ is synchronous. Therefore, a pattern can only form through a bifurcation, requiring an eigenvalue of $\textbf{J}$ with zero real part, which we refer to as a *critical eigenvalue* with corresponding *critical eigenvectors* and *critical eigenspace* (see Definition 8).

Consistent with our general assumptions, we assume that any admissible ODE of a regular network with *n* nodes takes the form

$$\begin{aligned} \dot{\textbf{x}}_{v_1}&= f(\textbf{x}_{v_1},\overline{\textbf{x}_{v^1_1},...,\textbf{x}_{v^1_{\nu }}},\lambda ) \\&\vdots \\ \dot{\textbf{x}}_{v_n}&= f(\textbf{x}_{v_n},\overline{\textbf{x}_{v^n_1},...,\textbf{x}_{v^n_{\nu }}},\lambda ) \end{aligned}$$

where $\lambda \in \mathbb {R}$ is a bifurcation parameter.

If we write the arguments of *f* as

$$\begin{aligned} f:= f(\textbf{u},\overline{\textbf{v}_1,...,\textbf{v}_{\nu }},\lambda ) \end{aligned}$$

and $(\textbf{x},\lambda )\in \Delta \times \mathbb {R}$, then the *internal and coupled dynamics* of the system (respectively) are given by

$$\begin{aligned} \textbf{Q}(\textbf{x},\lambda )&:= D_{\textbf{u}}f\big |_{(\textbf{x},\lambda )} \\ \textbf{R}(\textbf{x},\lambda )&:= D_{\textbf{v}_1}f\big |_{(\textbf{x},\lambda )} \ (= D_{\textbf{v}_2}f\big |_{(\textbf{x},\lambda )} =...=D_{\textbf{v}_{\nu }}f\big |_{(\textbf{x},\lambda )}) \end{aligned}$$

where $D_{\textbf{y}}f$ represents the differential of *f* with respect to the variable $\textbf{y} \in \mathbb {R}^s$. For example,

$$\begin{aligned} D_{\textbf{y}}f=\begin{pmatrix} \frac{\partial f_1}{\partial y_1} & \quad \frac{\partial f_1}{\partial y_2} & \quad ... & \quad \frac{\partial f_1}{\partial y_s} \\ \frac{\partial f_2}{\partial y_1} & \quad \frac{\partial f_2}{\partial y_2} & \quad ... & \quad \frac{\partial f_2}{\partial y_s} \\ \vdots & \quad \vdots & \quad \ddots & \quad \vdots \\ \frac{\partial f_s}{\partial y_1} & \quad \frac{\partial f_s}{\partial y_2} & \quad ... & \quad \frac{\partial f_s}{\partial y_s} \end{pmatrix}. \end{aligned}$$

We remove the arguments $(\textbf{x},\lambda )$ of $\textbf{Q}$ and $\textbf{R}$ if it is clear where they are being evaluated.

#### Definition 6

*(Adjacency Matrix)* The adjacency matrix of a regular network $\mathcal {N} =(\mathscr {V},\mathscr {A},s,t)$ with *n* nodes is a $n\times n$ matrix $\textbf{A}=(a_{ij})$ with entries $a_{ij}$ defined to be the number of arrows from node $v_j$ to node $v_i$ (i.e. $|\{a \in \mathscr {A}: s(a) = v_j, \ t(a) = v_i\}|$).

See Example 1.

#### Definition 7

*(Kronecker Product,* $\otimes$) Let $\textbf{B}=(b_{ij})$ and $\textbf{C}=(c_{ij})$ be matrices over $\mathbb {C}$ of arbitrary size. The Kronecker product of $\textbf{B}$ and $\textbf{C}$, denoted $\textbf{B}\otimes \textbf{C}$, is the block matrix given by

$$\begin{aligned} \textbf{B} \otimes \textbf{C} = (\textbf{B}c_{ij}). \end{aligned}$$

#### Proposition 2

(Eigenvalues of System via Reduced Matrices) Suppose $\mathcal {N}$ is a regular network with adjacency matrix $\textbf{A}$ and admissible ODE $\dot{\textbf{x}} = F(\textbf{x}, \lambda )$. If $\mu _1,...,\mu _k$ are the eigenvalues of $\textbf{A}$ (not necessarily distinct) with eigenvectors $\textbf{v}_1,...,\textbf{v}_k$, and $\textbf{J}= (dF)_{(\textbf{x},\lambda )}$ is the Jacobian of *F* evaluated at a synchronous equilibrium $(\textbf{x},\lambda ) \in \Delta \times \mathbb {R}$, then the eigenvalues of $\textbf{J}$ are the union of the eigenvalues of $\textbf{Q}+\mu _j \textbf{R}$ where $\textbf{Q}$ and $\textbf{R}$ are the internal and coupled dynamics, respectively. Furthermore, the corresponding eigenvectors are $\textbf{u} \otimes \textbf{v}_j$ where $\textbf{u}$ is an eigenvector of $\textbf{Q}+\mu _j \textbf{R}$.

#### Proof

See Golubitsky and Lauterbach (2009). $\square$

#### Example 1

Consider the network at the top of Fig. 2 (ignoring colors). Any admissible ODE has the form

$$\begin{aligned} \dot{\textbf{x}}_1&= f(\textbf{x}_1,\overline{\textbf{x}_1,\textbf{x}_2},\lambda ) \\ \dot{\textbf{x}}_2&= f(\textbf{x}_2,\overline{\textbf{x}_1,\textbf{x}_1},\lambda ) \\ \dot{\textbf{x}}_3&= f(\textbf{x}_3,\overline{\textbf{x}_1,\textbf{x}_2},\lambda ). \end{aligned}$$

For example, setting $f(u,\overline{v,w},\lambda )=-3u-2\lambda (v+w)$, the following is an admissible ODE with 1-dimensional node space.

$$\begin{aligned} \dot{x}_1=-3x_1 -2\lambda (x_1+x_2) \\ \dot{x}_2=-3x_2 - 2\lambda (x_1+x_1) \\ \dot{x}_3=-3x_3 - 2\lambda (x_1+x_2) \end{aligned}$$

Notice that this system has a synchronous equilibrium $(\textbf{0},\lambda )$ for all $\lambda$, and we can use Proposition 2 to compute the eigenvalues of the Jacobian at $(\textbf{0},\lambda )$. The network adjacency matrix is

$$\begin{aligned} \textbf{A} = \begin{pmatrix} 1 & \quad 1 & \quad 0 \\ 2 & \quad 0 & \quad 0 \\ 1 & \quad 1 & \quad 0 \end{pmatrix}, \end{aligned}$$

which has the following eigenvalues and eigenvectors.

$$\begin{aligned} \mu _1&=-1&v_1&= (1,-2,1)^T\\ \mu _2&=0&v_2&=(0,0,1)^T \\ \mu _3&=2&v_3&=(1,1,1)^T \end{aligned}$$

Since $D_u f (\textbf{0},\lambda )=-3$ and $D_v f(\textbf{0},\lambda )=-2\lambda$, by Proposition 2, the eigenvalues and eigenvectors of the system’s Jacobian $\textbf{J}=(dF)_{(\textbf{0},\lambda )}$ are

$$\begin{aligned}&-3 + 2\lambda &  (1,-2,1)^T \\&-3 &  (0,0,1)^T \\&-3-4\lambda &  (1,1,1)^T. \end{aligned}$$

### The critical eigenvalue dictates the preferred pattern

There may be bifurcations from a synchronous branch $(\textbf{x}(\lambda ),\lambda )$ that lead to new branches of solutions contained in $\Delta \times \mathbb {R}$; we call these *synchrony-preserving bifurcations*. Otherwise, they are *synchrony-breaking.* We assume that pattern formation occurs from a synchrony-breaking bifurcation and argue the first bifurcating pattern is the “preferred” pattern of a tissue. When the homogeneous steady state is destabilized via a bifurcation, the critical eigenvalue corresponds to a pattern (Theorems 3,4), which is generically the only stable bifurcating pattern (Proposition 5). We assume that the bifurcation parameter $\lambda$ changes much slower than the state of the cells. With a quasistatically (slowly) varying $\lambda$, the uniform state can lose stability with a stable patterned state emerging; thus, the system will move from the homogeneous state to the pattern state and will remain there unless there is a secondary bifurcation on the pattern branch. Furthermore, as in Poston and Stewart (1978), we may assume that the only bifurcations present are those that are forced by our assumptions and observed data. Extraneous secondary bifurcations would violate Occam’s Razor – the principle that the simplest model which can explain observed phenomena is best. Even with secondary bifurcations, the preferred pattern is the only stable pattern for some region of the parameter space, and it is reasonable that unusual behaviors can arise for unrealistic parameter values.

#### Definition 8

*(Eigenspace and Generalized Eigenspace)* Let $\textbf{A}$ be a real $n\times n$ matrix with eigenvalue $\mu$. The (real) eigenspace of $\mu$ is given by

$$\begin{aligned} P^{\mu }&= \text {ker}[\textbf{A}-\mu \textbf{I}] &  (\mu \in \mathbb {R}) \\ P^{\mu }&= \text {ker}[(\textbf{A}-\mu \textbf{I})(\textbf{A}-\overline{\mu }\textbf{I})] &  (\mu \in \mathbb {C} \setminus \mathbb {R}), \end{aligned}$$

and the (real) generalized eigenspace of $\mu$ is given by

$$\begin{aligned} G^{\mu }&= \text {ker}[(\textbf{A}-\mu \textbf{I})^n] &  (\mu \in \mathbb {R}) \\ G^{\mu }&= \text {ker}[(\textbf{A}-\mu \textbf{I})^n(\textbf{A}-\overline{\mu }\textbf{I})^n] &  (\mu \in \mathbb {C} \setminus \mathbb {R}). \end{aligned}$$

#### Theorem 3

(Steady State Patterns Bifurcating from Synchrony) Let $\mathcal {N}$ be a regular network with admissible ODE $\dot{\textbf{x}} = F(\textbf{x},\lambda )$. Let $\Delta$ denote the fully synchronous subspace, and let $\Delta _{\bowtie }\ne \Delta$ be the polysynchrony subspace associated with the balanced coloring $\bowtie$. Assume that there is a synchronous equilibrium $(\textbf{x}_0,\lambda _0)$ and that $\textbf{J}=(dF)_{(\textbf{x}_0,\lambda _0)}$ has a real critical eigenvalue $\mu$. Denote the associated generalized eigenspace $G^{\mu }$. If $G^{\mu } \cap \Delta = \{0\}$ and $\text {dim}(G^{\mu }\cap \Delta _{\bowtie })=1$, then generically a unique branch of equilibria with synchrony pattern $\bowtie$ bifurcates from $\textbf{x}_0$ at $\lambda _0$.

#### Proof (modified from Golubitsky and Stewart (2023), Theorem 18.10)

 Since $G^{\mu } \cap \Delta =\{0\}$, $\textbf{J}|_{\Delta }$ is nonsingular. Thus, by the implicit function theorem, there exists a synchronous branch of equilibria in the neighborhood of $(\textbf{x}_0,\lambda _0)$. By a change of coordinates, we can assume that the synchronous branch is given by $F(\textbf{0},\lambda )=0$ for $\lambda$ in a neighborhood of $\lambda _0$. Since $\text {dim}(G^{\mu } \cap \Delta _{\bowtie })=1$, the kernel of $\textbf{J}|_{\Delta _{\bowtie }}$ is 1-dimensional, so we can use Liapunov-Schmidt reduction to find a reduced equation of the restriction $F|_{\Delta _{\bowtie }}: \Delta _{\bowtie } \times \mathbb {R} \rightarrow \Delta _{\bowtie }$ given by

$$\begin{aligned} g: \text {span}_{\mathbb {R}}\{\textbf{v}\} \times \mathbb {R} \rightarrow \text {span}_{\mathbb {R}}\{\textbf{v}^*\}. \end{aligned}$$

for some vectors $\textbf{v},\textbf{v}^*$. The zeros of *g* are in one-to-one correspondence with the zeros of $F|_{\Delta _{\bowtie }}$ in a neighborhood of the bifurcation.

Since the spatial domain and codomain of *g* are 1-dimensional, we can write

$$\begin{aligned} g(s\textbf{v},\lambda ) = h(s,\lambda )\textbf{v}^* \end{aligned}$$

for $s\in \mathbb {R}$ and $h: \mathbb {R} \times \mathbb {R} \rightarrow \mathbb {R}$. Liapunov-Schmidt reduction can be chosen to preserve the existence of the trivial solution, so we may assume $h(0,\lambda )=0$.

With the generic assumption that $\frac{d}{d\lambda }\mu \big |_{\lambda =\lambda _0}\ne 0$ (the eigenvalue crossing condition), the reduction implies that $h_{\lambda }(0,\lambda _0)$ is generically nonzero. Thus, by the implicit function theorem $h(s(\lambda ),\lambda ))=0$ for some function *s* of $\lambda$. Since we can write *s* as a function of $\lambda$ in a neighborhood of the bifurcation, there exists a branch of solutions to $g=0$ and thus to $F|_{\Delta _{\bowtie }}=0$. $\square$

#### Remark 3

For the system given in Example 1, there is a bifurcation when $\lambda = 3/2$ that satisfyies the assumptions of Theorem 3. Namely, $G^{\mu } = \text {span}_{\mathbb {R}}\{(1,-2,1)^T\}$, so $G^{\mu }\cap \Delta = \{0\}$. Furthermore, for the balanced coloring $\bowtie$ shown in the top right of Fig. 2, $\text {dim}(G^{\mu }\cap \Delta _{\bowtie })=1$. Thus, a unique branch of equilibria with synchrony pattern $\bowtie$ generically bifurcates from the synchronous branch at $(\textbf{0},3/2)$.

#### Theorem 4

(Oscillating Patterns Bifurcating from Synchrony) Let $\mathcal {N}$ be a regular network with admissible ODE $\dot{\textbf{x}}=F(\textbf{x},\lambda )$ with a synchronous branch of equilibria $(\textbf{x}(\lambda ),\lambda )$. Suppose that $\textbf{J}=(dF)_{(\textbf{x}(\lambda _0),\lambda _0)}$ has purely imaginary eigenvalues $\pm i\omega$ for $\omega \ne 0$ and denote the associated (real) generalized eigenspace $G^{\pm i\omega }$. Furthermore, assume that

1. $$\dim (G^{\pm i\omega }\cap \Delta _{\bowtie })=2$$.
2. The eigenvalues cross the imaginary axis with nonzero speed.
3. There are no resonant eigenvalues $k\omega i$ with $k\in \mathbb {Z}, k\ne \pm 1$.

Then there exists a unique branch of periodic solutions bifurcating from synchrony with pattern $\bowtie$.

*Proof idea.* As in Golubitsky and Stewart (2023), Theorem 20.7, restrict *F* to $\Delta _{\bowtie }$ and apply the standard Hopf theorem.

#### Proposition 5

(Only the First Bifurcation is Stable) Suppose $\mathcal {N}$ is a regular network with admissible ODE $\dot{\textbf{x}}=F(\textbf{x},\lambda )$, and assume that there is a synchronous branch of equilibria $(\textbf{x}(\lambda ),\lambda )$ that is linearly stable for $\lambda <\lambda _0$ but loses stability as some eigenvalues of $\textbf{Q}+\mu _i \textbf{R}$ cross the imaginary axis. Any subsequent bifurcations from $(\textbf{x}(\lambda ),\lambda )$ cannot lead to stable branches so long as there is never a critical eigenvalue from $\textbf{Q}+\mu _i\textbf{R}$.

#### Proof

Let $\xi$ be an eigenvalue of $\textbf{Q}+\mu _i\textbf{R}$ (and hence the Jacobian by Proposition 2) that crosses the imaginary axis at $\lambda =\lambda _0$. Since for $\lambda >\lambda _0$, there is never a critical eigenvalue of $\textbf{Q}+\mu _i\textbf{R}$, $\xi$ will remain positive. Additionally, the eigenvalues of the Jacobian depend continuously on $(\textbf{x},\lambda )$, so the Jacobian evaluated on a bifurcating branch at some $\lambda >\lambda _0$ will have a positive eigenvalue in some neighborhood of the bifurcation point. $\square$

### Other preliminaries

In our results, we distinguish between *nondegenerate* and *degenerate* bifurcations. Nondegenerate bifurcations have a simple critical eigenvalue (i.e. a critical eigenvalue with algebraic multiplicity 1 or a single pair of purely imaginary eigenvalues), while degenerate bifurcations have critical eigenvalues with higher mutliplicities. To understand degeneracies, we will use the definition of a strongly connected network and the well-known Perron-Frobenius theorem.

#### Definition 9

*(Strongly Connected Network)* A network $\mathcal {N}$ is called strongly connected if for every pair of nodes $\{v_i,v_j\}$ with $i\ne j$, there is a path from $v_i$ to $v_j$ and from $v_j$ to $v_i$.

#### Theorem 6

(Perron-Frobenius) If a network $\mathcal {N}$ is strongly connected, then the adjacency matrix $\textbf{A}$ has a simple positive eigenvalue $\mu$ equal to the spectral radius $\rho (\textbf{A})$. For all other eigenvalues $\eta$ of $\textbf{A}$, $|\eta |<\rho (\textbf{A})$.

#### Proof

See Golubitsky and Stewart (2023), Theorem C.2. $\square$

#### Remark 4

We will use Perron-Frobenius twice:

1. In the proof of Theorem 10, we avoid degeneracies since the maximum eigenvalue is simple.
2. In Theorem 11, our conditions for a nondegenerate bifurcation depend on the spectral radius of $\textbf{A}$.

## Results

### The internal and coupled dynamics determine the preferred pattern

The adjacency matrix of the cell-communication network determines the possible patterns that can form in the tissue. Qualitative features of the internal and coupled dynamics select the preferred pattern from all possible ones.

Throughout this section, we make the following assumptions:

1. $\mathcal {N}=(\mathscr {V},\mathscr {A},s,t)$ is a regular network on *n* nodes (Definition 1) with adjacency matrix $\textbf{A}$ (Definition 6).
2. $\mathcal {N}$ has no self-arrows (i.e. for any $v\in \mathscr {V}$ there is no $a\in \mathscr {A}$ with $s(a)=v$ and $t(a)=v$).
3. $\dot{\textbf{x}} = F(\textbf{x},\lambda )$ is an arbitrary $\mathcal {N}$-admissible ODE with a synchronous branch of equilibria $(\textbf{x}(\lambda ),\lambda ) \in \Delta \times \mathbb {R}$ that is stable for $\lambda <\lambda _0$.
4. The Jacobian of *F* has an eigenvalue with zero real part when evaluated at $(\textbf{x}_0,\lambda _0) := (\textbf{x}(\lambda _0),\lambda _0) \in \Delta \times \mathbb {R}$.
5. $\textbf{Q}(\textbf{x},\lambda )$ and $\textbf{R}(\textbf{x},\lambda )$ are the linearized internal and coupled dynamics of *F* (respectively), which are defined whenever $(\textbf{x},\lambda ) \in \Delta \times \mathbb {R}$ (Section 2.3).
6. $\mathcal {N}$ is strongly connected (Definition 9).

We restate the relevant assumptions before each theorem for clarity.

We sometimes assume that the eigenvalues of $\textbf{A}$ are real – as we were not able to prove the complex case. The theory, however, still has wide applicability as many cell-communication networks have symmetric adjacency matrices, which have real eigenvalues.

**Fig. 3:**
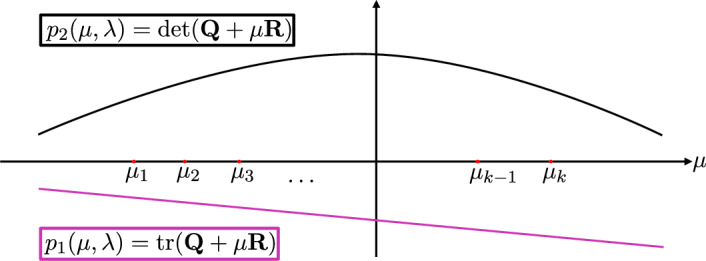
Illustration of the proofs for Theorems 10-13. Consider the trace and determinant of $\textbf{Q}+\mu \textbf{R}$ as polynomials in $\mu$ with $\textbf{A}$’s eigenvalues $\mu _1,...,\mu _k$ being points in the domain. By stability assumptions, $p_2(\mu _i)>0$ and $p_1(\mu _i)<0$ for all *i* when $\lambda <\lambda _0$. If $p_1$ or $p_2$ has a root that is some $\mu _i$, a bifurcation may occur. The trace is linear in $\mu$, and when $\det (\textbf{R})=0$, the determinant is linear. Assuming that one of the graphs intersects $(\mu _i,0)$ for some *i*, we can determine those *i* satisfying $p_1(\mu _i)=0$ or $p_2(\mu _i)=0$ from the slope of each line

Lastly, the intuition for most proofs comes from considering $\det (\textbf{Q}+\mu \textbf{R})$ and $\text {tr}(\textbf{Q}+\mu \textbf{R})$ as polynomials in $\mu$ with the eigenvalues of the adjacency matrix $\{\mu _i\}$ as points in the domain (Fig. 3). Varying the bifurcation parameter, these polynomials change smoothly until one has a root at some $\mu _i$, indicating that the system has a critical eigenvalue. Our theory provides criteria on the internal and coupled dynamics that restricts the possible roots $\mu _i$, and thus restricts the possible patterns that can form.

#### Definition 10

*(Critical Pattern Space)* Suppose that assumptions (A1), (A3)-(A5) hold. Furthermore, assume that $\textbf{A}$ has distinct eigenvalues $\mu _1,\mu _2,...,\mu _k$, and assume that $(dF)_{(\textbf{x}_0,\lambda _0)}$ has critical eigenvalues that by Proposition 2 are critical eigenvalues of $\{\textbf{Q}+\mu _j \textbf{R}\}_{j\in \mathcal {J}}$ for some $\mathcal {J}\subset \{1,2,...,k\}$. Then define the critical pattern space to be the subspace of $\mathbb {R}^n$ given by

$$\begin{aligned} \bigoplus _{j\in \mathcal {J}} P^{\mu _j}. \end{aligned}$$

where $P^{\mu _j}$ denotes the (real) eigenspace of $\mu _j$

#### Interpretation

Proposition 2 shows that critical eigenvectors have the form $\textbf{u}\otimes \textbf{v}$ where $\textbf{u}$ is an eigenvector of $\textbf{Q}+\mu _i\textbf{R}$ for some *i*, while $\textbf{v}$ is an eigenvector of the adjacency matrix $\textbf{A}$. Intuitively, $\textbf{u}$ is the part of the critical eigenvector inside each cell, while $\textbf{v}$ is the part on the level of the cell-communication network; therefore, to understand the pattern, it is important to consider the space spanned by $\textbf{v}$ for each critical eigenvector. This information is captured in the critical pattern space.

#### Proposition 7

(Stability of Synchronous 1-Dimensional Nodes) If the node space is 1-dimensional and the synchronous equilibrium $(\textbf{x}(\lambda ),\lambda )$ is stable, then $\textbf{Q}(\textbf{x}(\lambda ),\lambda )< 0$.

#### Lemma 7.1

For a regular network $\mathcal {N}$ without self arrows (assumptions (A1) and (A2)), the adjacency matrix $\textbf{A}$ has a strictly positive eigenvalue and an eigenvalue with strictly negative real part.

#### Proof of lemma

The adjacency matrix of a regular network always has a positive eigenvalue equal to its valence $\mu _k>0$ (see Definition 1). Furthermore, since we assume our graph has no self-arrows, every element on the diagonal of $\textbf{A}$ is zero, and $\text {tr}(\textbf{A})=0$. Since the trace is the sum of the eigenvalues and there exists a positive eigenvalue, there must be some eigenvalue with negative real part. $\square$

#### Proof of proposition

Recall that the eigenvalues of the system are $\textbf{Q}+\mu _i \textbf{R}$ where $\mu _i$ are the eigenvalues of the adjacency matrix $\textbf{A}$ (Proposition 2). By Lemma 7.1, $\textbf{A}$ has a positive eigenvalue $\mu _k$ and an eigenvalue $\mu _1$ with negative real part. Let each eigenvalue be written

$$\begin{aligned} \mu _j=u_j+\iota v_j \end{aligned}$$

where $\iota ^2=-1$. For the remainder of this proof, we will drop the arguments of the internal and coupled dynamics, $\textbf{Q}$ and $\textbf{R}$ (respectively), writing

$$\begin{aligned} \textbf{Q}&:= \textbf{Q}(\textbf{x}(\lambda ),\lambda )\\ \textbf{R}&:=\textbf{R}(\textbf{x}(\lambda ),\lambda ). \end{aligned}$$

Since $(\textbf{x}(\lambda ),\lambda )$ is stable, the real part of each eigenvalue $\textbf{Q}+u_i\textbf{R}<0$ for all *i*. By Lemma 7.1, we can choose $\mu _j$ such that $\text {sgn}(u_j) = \text {sgn}(\textbf{R})$. Then

$$\begin{aligned}&\textbf{Q}+u_j \textbf{R} \\&\quad = \textbf{Q}+|u_j \textbf{R}|<0 \\&\quad \implies \textbf{Q}<-|u_j \textbf{R}| \le 0. \ \end{aligned}$$

$$\square $$

#### Proposition 8

(Stability of Synchronous 2-dimensional Nodes) Assume (A1)-(A5), and let the node space be 2-dimensional. If the synchronous branch $(\textbf{x}(\lambda ),\lambda )$ is stable, then $\text {tr}(\textbf{Q}(\textbf{x}(\lambda ),\lambda ))<0$.

#### Proof

As before, let each eigenvalue be written

$$\begin{aligned} \mu _j=u_j+\iota v_j \end{aligned}$$

where $\iota ^2=-1$. Since $(\textbf{x}(\lambda ),\lambda )$ is stable,

$$\begin{aligned} \text {Re}(\text {tr}(\textbf{Q}+\mu _i\textbf{R}))=\text {tr}(\textbf{Q}+u_i \textbf{R})<0 \end{aligned}$$

for $1\le i\le k$. By Lemma 7.1, there exists eigenvalues of $\textbf{A}$ with positive and negative real part. Let $\mu _j$ be an eigenvalue whose real part has the same sign as $\text {tr}(\textbf{R})$. Then

$$\begin{aligned} \text {tr}(\textbf{Q}+u_j \textbf{R})&= \text {tr}(\textbf{Q}) + u_j\text {tr}(\textbf{R}) \\&= \text {tr}(\textbf{Q}) + |u_j\text {tr}(\textbf{R})|<0 \\&\implies \text {tr}(\textbf{Q})<-|u_j \text {tr}(\textbf{R})| \le 0. \\ \end{aligned}$$

$$\square $$

#### Theorem 9

(Characterizing the First Branch in 1-Dimension) Assume (A1)-(A5). Let $\textbf{A}$ have eigenvalues $\mu _1,...,\mu _k$ ordered by real part, and assume that the node space is 1-dimensional. Then we have the following mutually exclusive cases

1. The critical pattern space is $\mathbb {R}^n$ if and only if $\textbf{R}(x_0,\lambda _0)=0$.
2. The critical pattern space is $P^{\mu _1}$ if and only if $\textbf{R}(x_0,\lambda _0)<0$.
3. The critical pattern space is $P^{\mu _k} = \text {span}_{\mathbb {R}}\{\vec {1}_n\}$ (a vector of ones of length *n*) if and only if $\textbf{R}(x_0,\lambda _0)>0$.

#### Remark 5

Nondegenerate bifurcations with critical pattern space $P^{\mu _k}$ are synchrony-preserving, while those with critical pattern space $P^{\mu _1}$ are synchrony-breaking.

#### Interpretation

Write each eigenvalue as $\mu _i = u_i + \iota v_i$ where $\iota ^2=-1$; then the real part of each eigenvalue is given by $\textbf{Q}+u_i \textbf{R}$. Theorem 9 uses the intuition that $p(u):=\textbf{Q}+u \textbf{R}$ is a linear function of *u* (see Fig. 3). Since the synchronous equilibrium is initially stable, $p(u_i)<0$ for every *i*. Thus, the graph $\{(u,p(u))\}$ is a line lying below the *x*-axis $\{(u,0)\}$ for *u* in the interval $[u_1,u_k]$. A bifurcation can occur when $p(u_i)=0$ for some *i*, but the graph of a smoothly changing line, $\{(u,p(u))\}$, can only intersect the set of points $\{(u_1,0),...,(u_k,0)\}$ in three mutually exclusive ways: with positive slope, negative slope, or zero slope. These are described rigorously in Lemma 9.1 below and lead to the three cases in Theorem 9. Similar intuition is used to outline the cases in Theorem 10.

#### Lemma 9.1

Suppose that $p(\mu ,\lambda ): \mathbb {R} \times \mathbb {R} \rightarrow \mathbb {R}$ is a polynomial in $\mu$ smoothly parameterized by $\lambda$. Let $\mu _1<\mu _2<...<\mu _k$ be a set of points in $\mathbb {R}$. Let $\delta >0$, and suppose that for all $\lambda \in (\lambda _0-\delta ,\lambda _0)$ we have $p(\mu _i,\lambda )<0$ for all *i*. Suppose that at $\lambda _0$, $p(\mu ,\lambda _0)=\alpha (\lambda _0)+\beta (\lambda _0)\mu$ is linear in $\mu$ and there exists some *j* such that $p(\mu _j,\lambda _0)=0$. Then we have the following cases

1. $p(\mu _i,\lambda _0)=0$ for all *i* if and only if $\beta (\lambda _0)=0$.
2. $p(\mu _1,\lambda _0)=0$ and $p(\mu _i,\lambda _0)<0 \ \forall i\ne 1$ if and only if $\beta (\lambda _0)<0$.
3. $p(\mu _k,\lambda _0)=0$ and $p(\mu _i,\lambda _0)<0 \ \forall i\ne k$ if and only if $\beta (\lambda _0)>0$.

#### Proof of lemma

For the forward direction of (1), if $p(\mu _i,\lambda _0)=0$ for all *i*, then $p(\cdot ,\lambda _0) \equiv 0$ since it is a linear function of $\mu$; therefore, $\beta (\lambda _0)=0$. For the converse, suppose that $\beta (\lambda _0)=0$. Then $p(\mu ,\lambda _0) = \alpha (\lambda _0)$. By hypothesis, there exists some $\mu _j$ such that $p(\mu _j,\lambda _0) = \alpha (\lambda _0)=0$. Clearly, this implies that $\alpha (\lambda _0)=0$. Thus, $p(\cdot ,\lambda _0) \equiv 0$, so $p(\mu _i,\lambda _0)=0$ for all *i*.

For the forward direction of (2), suppose that $p(\mu _1,\lambda _0)=0$ and $p(\mu _i,\lambda _0)<0$ for all $i\ne 1$. This implies that for any $i\ne 1$,

$$\begin{aligned} \alpha (\lambda _0) +\beta (\lambda _0) \mu _i < \alpha (\lambda _0)+\beta (\lambda _0)\mu _1. \end{aligned}$$

Equivalently,

$$\begin{aligned} \beta (\lambda _0)\mu _i < \beta (\lambda _0)\mu _1. \end{aligned}$$

Since $\mu _1 < \mu _i$, we must have that $\beta (\lambda _0)<0$.

For the converse, suppose that $\beta (\lambda _0)<0$. If

$$\begin{aligned} p(\mu _j,\lambda _0)=\alpha (\lambda _0)+\beta (\lambda _0)\mu _j =0 \text { for some }j\ne 1, \end{aligned}$$

then since $$\mu _1 < \mu _j$$,

$$\begin{aligned} p(\mu _1,\lambda _0) = \alpha (\lambda _0)+\beta (\lambda _0)\mu _1 >0, \end{aligned}$$

but this contradicts the continuity of $p(\mu ,\lambda )$ with respect to $\lambda$ since $p(\mu _1,\lambda )<0$ for $\lambda \in (\lambda _0-\delta ,\lambda _0)$. Hence, $\mu _1$ must be the unique point from $\{\mu _1,...,\mu _k\}$ with $p(\mu _1,\lambda _0)=0$.

For the forward direction of (3), suppose that $p(\mu _k,\lambda _0)=0$ and $p(\mu _i,\lambda _0)<0$ for all $i\ne k$. This implies that for any $i\ne k$,

$$\begin{aligned} \alpha (\lambda _0) +\beta (\lambda _0) \mu _i < \alpha (\lambda _0)+\beta (\lambda _0)\mu _k. \end{aligned}$$

Equivalently,

$$\begin{aligned} \beta (\lambda _0)\mu _i < \beta (\lambda _0)\mu _k. \end{aligned}$$

Since $\mu _i < \mu _k$, we must have that $\beta (\lambda _0)>0$.

For the converse, suppose that $\beta (\lambda _0)>0$. If

$$\begin{aligned} p(\mu _j,\lambda _0)=\alpha (\lambda _0)+\beta (\lambda _0)\mu _j =0 \text { for some }j\ne k, \end{aligned}$$

then since $$\mu _j < \mu _k$$,

$$\begin{aligned} p(\mu _1,\lambda _0) = \alpha (\lambda _0)+\beta (\lambda _0)\mu _k >0, \end{aligned}$$

but this contradicts the continuity of $p(\mu ,\lambda )$ with respect to $\lambda$ since $p(\mu _k,\lambda )<0$ for $\lambda \in (\lambda _0-\delta ,\lambda _0)$. Hence, $\mu _k$ must be the unique point from $\{\mu _1,...,\mu _k\}$ with $p(\mu _k,\lambda _0)=0$. $\square$

#### Proof of theorem

Let $\mu _1,\mu _2,...,\mu _k$ be the eigenvalues of $\textbf{A}$, ordered by their real parts, $u_1,u_2,...,u_k$. Define $p(u,\lambda )$ to be the real valued linear polynomial in *u* given by

$$\begin{aligned} p(u,\lambda )=\textbf{Q}(\textbf{x}(\lambda ),\lambda )+u\textbf{R}(\textbf{x}(\lambda ),\lambda ), \end{aligned}$$

so that $p(u_i,\lambda )$ gives the real part of $\textbf{Q}+\mu _i\textbf{R}$, which is an eigenvalue of $\textbf{J}$ by Proposition 2. Since we assume that the synchronous branch is stable before the bifurcation, there exists $\delta >0$ such that for all $\lambda \in (\lambda _0-\delta ,\lambda _0)$, $p(u_i,\lambda )<0$ for all *i*. Furthermore, at the bifurcation point, there exists some $u_j$ with $p(u_j,\lambda _0)=0$. Therefore, the hypotheses of Lemma 9.1 are satisfied.

Applying Lemma 9.1, we have the following three cases.

1. $p(u_i,\lambda _0)=0$ for all *i* if and only if $\textbf{R}(\textbf{x}(\lambda _0),\lambda _0)=0$.
2. $p(u_1,\lambda _0)=0$ and $p(u_i,\lambda _0)<0 \ \forall i\ne 1$ if and only if $\textbf{R}(\textbf{x}(\lambda _0),\lambda _0)<0$.
3. $p(u_k,\lambda _0)=0$ and $p(u_i,\lambda _0)<0 \ \forall i\ne k$ if and only if $\textbf{R}(\textbf{x}(\lambda _0),\lambda _0)>0$.

$p(u_j)=0$ exactly when the system has the critical eigenvalue $\textbf{Q}+\mu _j\textbf{R}$. Using Definition 10 the main conclusion immediately follows.

Lastly, the network $\mathcal {N}$ is strongly-connected, so Perron-Frobenius (Theorem 6) implies that there is a unique eigenvalue of $\textbf{A}$ with largest real part $\mu _k$. For regular networks, $\mu _k$ is equal to the valence of the network and has the eigenvector $\textbf{v}_k = \vec {1}$ (a vector of ones), so $P^{\mu _k}=\text {span}_{\mathbb {R}}\{\vec {1}_n\}$ by definition of the critical pattern space. $\square$

#### Theorem 10

(Characterization of Bifurcations in 2-dimensions with $\det (\textbf{R})=0$) Assume (A1)-(A6). Furthermore, suppose that the node space is 2-dimensional and the adjacency matrix $\textbf{A}$ has real eigenvalues $\mu _1<\mu _2<...<\mu _k$, where each $\mu _i$ has algebraic multiplicity $\alpha _i$. Suppose that $\det (\textbf{R}(\textbf{x}_0,\lambda _0))=0$, and take

$$\begin{aligned} B(\textbf{x}_0,\lambda _0):=\text {tr}(\textbf{Q}(\textbf{x}_0,\lambda _0))\text {tr}(\textbf{R}(\textbf{x}_0,\lambda _0)) - \text {tr}(\textbf{Q}(\textbf{x}_0,\lambda _0)\textbf{R}(\textbf{x}_0,\lambda _0)). \end{aligned}$$

In Table 1, we enumerate all possible critical pattern spaces and the multiplicity and type of critical eigenvalues. For the first four cases listed, we give necessary and sufficient conditions on the internal and coupled dynamics for such a bifurcation to occur. For the remaining cases, we give necessary conditions, which are sufficient if we assume the bifurcation is sufficiently degenerate.

**Table 1:** Enumeration of all critical pattern spaces, and the multiplicity and type of critical eigenvalues, given certain conditions on the internal and coupled dynamics. The condition “NDG" stands for nondegeneracy and refers to any condition on the trace or determinant of $\textbf{Q}+\mu _i \textbf{R}$ that ensures it is nonzero for every *i* (therefore, reducing the number of critical eigenvalues). An example of such conditions is given in Theorem 11. If $\alpha _1 = 1$, the first four bifurcations have simple critical eigenvalues (see Corollary 11.1) and thus generically lead to steady state or Hopf bifurcations with critical pattern spaces $P^{\mu _1}$ (synchrony-breaking) or $P^{\mu _k}$ (synchrony-preserving).

Critical Pattern Space	Critical Eigenvalues	Determinant Condition	Trace Condition
$P^{\mu _1}$	$\alpha _1$ (real)	$B>0$	NDG
$P^{\mu _1}$	$2\alpha _1$ (imag.)	NDG	$\text {tr}(\textbf{R})<0$
$P^{\mu _k}$	1 (real)	$B<0$	NDG
$P^{\mu _k}$	2 (imag.)	NDG	$\text {tr}(\textbf{R})$ > 0
$P^{\mu _1}$	$2\alpha _1$ (real)	$B>0$	$\text {tr}(\textbf{R})<0$
$P^{\mu _k}$	2 (real)	$B<0$	$\text {tr}(\textbf{R})>0$
$P^{\mu _1}\oplus P^{\mu _k}$	$\alpha _1$ (real)	$B>0$	$\text {tr}(\textbf{R})>0$
	2 (imag.)		
$P^{\mu _1}\oplus P^{\mu _k}$	2$\alpha _1$ (imag.)	$B<0$	$\text {tr}(\textbf{R})<0$
	1 (real)		
$\mathbb {R}^n$	$2\alpha _1$ (real)	$B>0$	$\text {tr}(\textbf{R})=0$
	$$\sum _{j\ne 1}2\alpha _j$$ (imag.)		
$\mathbb {R}^n$	2 (real)	$B<0$	$\text {tr}(\textbf{R})=0$
	$$\sum _{j\ne k}2\alpha _j$$ (imag.)		
$\mathbb {R}^n$	$2\alpha _1$ (real)	$B=0$	$\text {tr}(\textbf{R})<0$
	$$\sum _{j\ne 1}\alpha _j$$ (real)		
$\mathbb {R}^n$	2 (real)	$B=0$	$\text {tr}(\textbf{R})>0$
	$$\sum _{j\ne k}\alpha _j$$ (real)		
$\mathbb {R}^n$	$\sum _{1\le j \le k}2\alpha _j$ (imag.)	NDG	$\text {tr}(\textbf{R})=0$
$\mathbb {R}^n$	$\sum _{1\le j\le k}\alpha _j$ (real)	$B=0$	NDG
$\mathbb {R}^n$	$\sum _{1\le j\le k}2\alpha _j$ (real)	$B=0$	$\text {tr}(\textbf{R})=0$

#### Remark 6

The assumption that $\det (\textbf{R}) = 0$ is satisfied in many models of cell communication, including the model by Collier et al. (1996). Intuitively, this condition reflects the sparsity of intercellular chemical signaling compared to intracellular interactions; however, a deeper investigation of this relationship remains a subject for future work.

#### Proof of theorem

We want to use the trace and determinant to obtain information about the eigenvalues; therefore, define the following polynomials of $\mu$:

$$\begin{aligned} p_1(\mu ,\lambda )&= \text {tr}(\textbf{Q}(\textbf{x}(\lambda ),\lambda )+\mu \textbf{R}(\textbf{x}(\lambda ),\lambda )) \\&= \text {tr}((\textbf{Q}(\textbf{x}(\lambda ),\lambda )) + \mu \text {tr}(\textbf{R}(\textbf{x}(\lambda ),\lambda ))\\ p_2(\mu ,\lambda )&= \det (\textbf{Q}(\textbf{x}(\lambda ),\lambda )+\mu \textbf{R}(\textbf{x}(\lambda ),\lambda )) \\ \end{aligned}$$

We will drop the argument $\lambda$ if it is obvious where the polynomial is being evaluated. Since we assume that $\det (\textbf{R}(\textbf{x}_0,\lambda _0))=0$, we have that

$$\begin{aligned} p_2(\mu ,\lambda _0)&=[\text {tr}(\textbf{Q}(\textbf{x}_0,\lambda _0)\text {tr}(\textbf{R}(\textbf{x},\lambda )) - \text {tr}(\textbf{Q}(\textbf{x}_0,\lambda _0)\textbf{R}(\textbf{x}_0,\lambda _0))]\mu + \det (\textbf{Q}(\textbf{x}_0,\lambda _0)) \\&= \det (\textbf{Q}(\textbf{x}_0,\lambda _0))+\mu \textbf{B}(\textbf{x}_0,\lambda _0) \end{aligned}$$

We also assume the synchronous equilibrium is initially stable, so there exists $\delta >0$ such that for all $\lambda \in (\lambda _0-\delta ,\lambda _0)$,

1 $$\begin{aligned} p_1(\mu _i)<0 \end{aligned}$$

2 $$\begin{aligned} p_2(\mu _i)>0 \end{aligned}$$

for all *i*. This setup is illustrated in Fig. 3. For a bifurcation to happen, there exists $\mu _j$ such that either $p_1(\mu _j)=0$ or $p_2(\mu _j)=0$ (or both). Applying lemma 3.1.2 (to $p_1$ and $-p_2$), we have that

1. $p_1(\mu _1)=0$ and $p_1(\mu _i)<0 \ \forall i\ne 1$ if and only if $\text {tr}(\textbf{R})<0$.
2. $p_1(\mu _k)=0$ and $p_1(\mu _i)<0 \ \forall i\ne k$ if and only if $\text {tr}(\textbf{R})>0$.
3. $p_2(\mu _1)=0$ and $p_2(\mu _i)>0 \ \forall i\ne 1$ if and only if $B>0$.
4. $p_2(\mu _k)=0$ and $p_2(\mu _i)>0 \ \forall i\ne k$ if and only if $B<0$.
5. $p_1(\mu _i) = 0$ for all *i* if and only if $\text {tr}(\textbf{R})=0$.
6. $p_2(\mu _i) = 0$ for all *i* if and only if $B=0$.

Now, let’s consider some of the possible cases. Notice that by equations (1) and (2) and the continuity of both polynomials with respect to $\lambda$, if $p_1(\mu _j,\lambda _0)\ne 0$, then we must have $p_1(\mu _j,\lambda _0)<0$. Similarly, if $p_2(\mu _j,\lambda _0)\ne 0$, then we must have $p_2(\mu _j,\lambda _0)>0$.

If $p_1(\mu _i) =0$ and $p_2(\mu _i)>0$, there are $\alpha _i$ complex conjugate pairs critical eigenvalues that are eigenvalues of $\textbf{Q}(\textbf{x}_0,\lambda _0)+\mu _i \textbf{R}(\textbf{x}_0,\lambda _0)$; if $p_2(\mu _i)=0$ and $p_1(\mu _i)>0$, then there are $\alpha _i$ real critical eigenvalues that are an eigenvalue of $\textbf{Q}(\textbf{x}_0,\lambda _0)+\mu _i\textbf{R}(\textbf{x}_0,\lambda _0)$; and if $p_1(\mu _i)=p_2(\mu _i)=0$, there are $2\alpha _i$ real critical eigenvalues that are the two eigenvalues of $\textbf{Q}(\textbf{x}_0,\lambda _0)+\mu _i\textbf{R}(\textbf{x}_0,\lambda _0)$ with multiplicity $\alpha _i$.

Lastly, by the Perron-Frobenius Theorem, $\alpha _k=1$. We use this information to enumerate all possibilities in Table 1. $\square$

#### Theorem 11

(Nondegeneracy Conditions in 2-Dimensions) Assume (A1)–(A6) and that $\mathcal {N}$ has valence $\nu$. Suppose that the node space is 2-dimensional and the adjacency matrix $\textbf{A}$ has distinct real eigenvalues $\mu _1<\mu _2<...<\mu _k$ and corresponding algebraic multiplicities $\alpha _1,\alpha _2,...,\alpha _k$. Suppose that $\det (\textbf{R}(\textbf{x}_0,\lambda _0))=0$ and take

$$\begin{aligned} B:=\text {tr}(\textbf{Q}(\textbf{x}_0,\lambda _0))\text {tr}(\textbf{R}(\textbf{x}_0,\lambda _0)) - \text {tr}(\textbf{Q}(\textbf{x}_0,\lambda _0)\textbf{R}(\textbf{x}_0,\lambda _0)). \end{aligned}$$

1. Suppose that at $(\textbf{x}_0,\lambda _0)$, $(\text {tr}(\textbf{Q})\ne 0)\vee (\text {tr}(\textbf{R})\ne 0)$ where $\vee$ denotes the logical “or.” If
  $$\begin{aligned} \det (\textbf{Q})&> |\nu B|, \\ \end{aligned}$$
  then the critical eigenvalues are the pair of imaginary eigenvalues from a single matrix $\textbf{Q}+\mu _j \textbf{R}$, with multiplicity $\alpha _j$.
2. Suppose that at $(\textbf{x}_0,\lambda _0)$, $(B \ne 0)\vee (\det (\textbf{Q})\ne 0)$. If
  $$\begin{aligned} \text {tr}(\textbf{Q}) < -|\nu \text {tr}(\textbf{R})|, \end{aligned}$$
  then the critical eigenvalues are a real eigenvalue from a single matrix $\textbf{Q}+\mu _j \textbf{R}$ with multiplicity $\alpha _j$.

#### Proof

Since we can obtain important information about the eigenvalues of a $2\times 2$ matrix using the trace and determinant, define the following polynomials of $\mu$:

$$\begin{aligned} p_1(\mu ,\lambda )&= \text {tr}(\textbf{Q}(\textbf{x}(\lambda ),\lambda )+\mu \textbf{R}(\textbf{x}(\lambda ),\lambda ) \\&= \text {tr}((\textbf{Q}(\textbf{x}(\lambda ),\lambda )) + \mu \text {tr}(\textbf{R}(\textbf{x}(\lambda ),\lambda ))\\ p_2(\mu ,\lambda )&= \det (\textbf{Q}(\textbf{x}(\lambda ),\lambda )+\mu \textbf{R}(\textbf{x}(\lambda ),\lambda )) .\\ \end{aligned}$$

Again, this setup is illustrated in Fig. 3. We will drop the arguments $\lambda$ if it is obvious where the polynomial is being evaluated. Since we assume that $\det (\textbf{R}(\textbf{x}_0,\lambda _0))=0$, we have that

$$\begin{aligned} p_2(\mu ,\lambda _0)&=[\text {tr}(\textbf{Q}(\textbf{x}_0,\lambda _0)\text {tr}(\textbf{R}(\textbf{x}_0,\lambda _0)) - \text {tr}(\textbf{Q}(\textbf{x}_0,\lambda _0)\textbf{R}(\textbf{x}_0,\lambda _0))]\mu + \det (\textbf{Q}(\textbf{x}_0,\lambda _0)) \\&= \det (\textbf{Q}(\textbf{x}_0,\lambda _0))+\mu B(\textbf{x}_0,\lambda _0). \end{aligned}$$

Suppose that $\det (\textbf{Q})> |\nu B|$ at the bifurcation. Of course, this implies that $\det (\textbf{Q})>0$. Furthermore, by the Perron-Frobenius theorem $\nu$ is the spectral radius of $\textbf{A}$ (i.e. the largest absolute value of its eigenvalues). Therefore, for any *i*,

$$\begin{aligned}&\det (\textbf{Q})>|\nu B| \\ \implies&\det (\textbf{Q}) > |\mu _i B|, \end{aligned}$$

and

$$\begin{aligned} p_2(\mu _i) = \det (\textbf{Q})+\mu _iB > 0. \end{aligned}$$

Since $\det (\textbf{Q}+\mu _i\textbf{R})>0$ for all *i*, the critical eigenvalues must correspond to pairs of imaginary eigenvalues, given by matrices $\textbf{Q}+\mu _i \textbf{R}$ such that $p_1(\mu _i)=0$. In the case that $\text {tr}(\textbf{Q})\ne 0$, there is exactly one root of the line $p_1$, implying that at the bifurcation, there is a unique $\mu _j$ such that $p_1(\mu _j)=0$. Thus, there is a pair of imaginary critical eigenvalues that are eigenvalues of $\textbf{Q}+\mu _j\textbf{R}$. Since $\mu _j$ is an eigenvalue of $\textbf{A}$ with multiplicity $\alpha _j$, the critical eigenvalue is an eigenvalue of $\textbf{J}=(dF)_{(\textbf{x}_0,\lambda _0)}$ with multiplicity $\alpha _j$. Now, notice that $\text {tr}(\textbf{R})\ne 0$ implies $\text {tr}(\textbf{Q})<0$ (otherwise the synchronous branch would be unstable before the bifurcation), and thus our analysis of the case that $\text {tr}(\textbf{R})\ne 0$ reduces to the previous case that $\text {tr}(\textbf{Q})\ne 0$.

For (2), suppose that $\text {tr}(\textbf{Q})<-|\nu \text {tr}(\textbf{R})|$ at the bifurcation. Then $\text {tr}(\textbf{Q})<0$. Furthermore, for any *i*,

$$\begin{aligned}&\text {tr}(\textbf{Q})<-|\nu \text {tr}(\textbf{R})| \\ \implies&\text {tr}(\textbf{Q}) < -|\mu _i\text {tr}(\textbf{R})|, \end{aligned}$$

and

$$\begin{aligned} p_1(\mu _i) = \text {tr}(\textbf{Q})+\mu _i \text {tr}(\textbf{R}) < 0. \end{aligned}$$

Since $p_1(\mu _i)<0$ for all *i* at the bifurcation, any critical eigenvalues must be real and given by matrices $\textbf{Q}+\mu _i\textbf{R}$ with $p_2(\mu _i)=0$. Using the same logic as previously, if $\det (\textbf{Q})\ne 0$, there can be exactly one root of the line $p_2$, implying that at the bifurcation there is a unique $\mu _j$ with $p_2(\mu _j)=0$, giving a real critical eigenvalue that is an eigenvalue of $\textbf{Q}+\mu _j \textbf{R}$. Since $\mu _j$ is an eigenvalue of $\textbf{A}$ with multiplicity $\mu _j$, the critical eigenvalue is an eigenvalue of $\textbf{J}$ with multiplicity $\alpha _j$. We also have that $B\ne 0$ implies that $\det (\textbf{Q})> 0$ (otherwise the synchronous branch would be unstable before the bifurcation), thus reducing to the prior case. In both cases, since $\text {tr}(\textbf{Q}+\mu _j\textbf{R})<0$ and $\det (\textbf{Q}+\mu _j\textbf{R})=0$, the critical eigenvalue is real. $\square$

#### Corollary 11.1

Assume (A1)-(A6). Furthermore, suppose that the node space is 2-dimensional and the adjacency matrix $\textbf{A}$ has real eigenvalues $\mu _1<\mu _2<...<\mu _k$. If there is a nondegenerate synchrony-breaking bifurcation, then the critical pattern space is $P^{\mu _1}$.

#### Proof of corollary

Note that the hypotheses of Theorem 10 are satisfied. Referencing the second column of Table 1, a nondegenerate bifurcation (with simple critical eigenvalue) can only occur under the conditions given in rows 1-4. In these rows, the critical pattern space is either $P^{\mu _1}$ or $P^{\mu _k}$; but since we assume the bifurcation is synchrony-breaking, the critical pattern space cannot be $P^{\mu _k}$. Indeed, if the critical pattern space is $P^{\mu _k} = \text {span}_{\mathbb {R}}\{\vec {1}_n\}$, then the critical generalized eigenspace $G^{\mu }\subset \Delta$, implying that any bifurcating solutions will be contained in $\Delta$ (i.e. a synchrony-preserving bifurcation). In conclusion, the critical pattern space must be $P^{\mu _1}$. $\square$

In contrast to Corollary 11.1, if $\det (\textbf{R})\ne 0$ then a stable pattern can correspond to any critical pattern space $P^{\mu _i}$ as shown below. This indicates two possible mechanisms for the diversity of patterns we see in biological systems: cells reuse chemical signaling pathways but reorganize themselves to change communication, or cells use different chemical signaling pathways.

#### Theorem 12

(Existence of Admissible ODE with Arbitrary First Bifurcation, Golubitsky and Stewart) Assume (A1)-(A6), and suppose the adjacency matrix $\textbf{A}$ has distinct real eigenvalues $\mu _1<...<\mu _k$. Then for any *i*, there exists an admissible system with 2-dimensional node space such that the critical pattern space is $P^{\mu _i}$.

#### Proof

See Golubitsky and Stewart (2023), Theorem 18.22. $\square$

#### Theorem 13

(Sufficient Condition for Pattern in $P^{\mu _1}$ or $P^{\mu _k}$) Assume (A1)-(A6). Furthermore, suppose that the node space is 2-dimensional and the adjacency matrix $\textbf{A}$ has distinct real eigenvalues $\mu _1<\mu _2<...<\mu _k$ not counting multiplicity. If $\det (\textbf{R}(\textbf{x}_0,\lambda _0))\le 0$, then the critical pattern space is either $P^{\mu _1}$, $P^{\mu _k}$, $P^{\mu _1}\oplus P^{\mu _k}$, or $\mathbb {R}^n$.

#### Proof

As before, let

$$\begin{aligned} p_1(\mu ,\lambda )&= \text {tr}(\textbf{Q}(\textbf{x}(\lambda ),\lambda )+\mu \textbf{R}(\textbf{x}(\lambda ),\lambda ) \\&= \text {tr}((\textbf{Q}(\textbf{x}(\lambda ),\lambda )) + \mu \text {tr}(\textbf{R}(\textbf{x}(\lambda ),\lambda ))\\ p_2(\mu ,\lambda )&= \det (\textbf{Q}(\textbf{x}(\lambda ),\lambda )+\mu \textbf{R}(\textbf{x}(\lambda ),\lambda )). \end{aligned}$$

If $p_1(\cdot ,\lambda _0) \equiv 0$ or $p_2(\cdot ,\lambda _0) \equiv 0$, then there are critical eigenvalues from $\textbf{Q}+\mu _i \textbf{R}$ for all *i*, and the critical pattern space is $\mathbb {R}^n$.

Now suppose that $p_1 \not \equiv 0$ and $p_2 \not \equiv 0$. Then, there is a critical eigenvalue that is an eigenvalue of $\textbf{Q}+\mu _i \textbf{R}$ if and only if either $p_1(\mu _i,\lambda _0)=0$ or $p_2(\mu _i,\lambda _0)=0$. From Lemma 9.1, if $p_1(\mu _i,\lambda _0)=0$ then $i=1,k$.

$p_2(\mu ,\lambda _0)$ is a quadratic polynomial in $\mu$ whose graph is downward opening (Fig. 3), so its derivative is linear with slope $2\det (\textbf{R}(\textbf{x}_0,\lambda _0))<0$. Suppose that $p_2(\mu _i,\lambda _0)=0$ for $i\ne 1,k$ for contradiction. We have two cases:

1. If $\frac{\partial }{\partial \mu } p_2\big |_{(\mu _i,\lambda _0)}>0$, then $p_2(\mu _1,\lambda _0)<0$ since $\mu _1<\mu _i$. But this contradicts the continuity of $p_2$ in $\lambda$ since $p_2(\mu _i,\lambda _0)>0$ for $\lambda <\lambda _0$, otherwise the branch $(\textbf{x}(\lambda ),\lambda )$ would not be stable.
2. Similarly, if $\frac{\partial }{\partial \mu }p_2\big |_{(\mu _i,\lambda _0)}<0$, then $p_2(\mu _k,\lambda _0)<0$ since $\mu _k>\mu _i$, which again contradicts the continuity of $p_2$ as a function of $\lambda$.

$$\square $$

In conclusion, since $p_1(\mu _i)=0$ or $p_2(\mu _j)=0$ can only occur for $i=1,k$ or $j=1,k$, there can only be critical eigenvalues from $\textbf{Q}+\mu _1\textbf{R}$ or $\textbf{Q}+\mu _k\textbf{R}$ (or both), which gives us our conclusion by Proposition 2 and Definition 10.

### Predicting patterns from qualitative features of cell-communication and chemical kinetics

We use theorems in Sect. 3.1 to predict patterns of cell fates given qualitative features of Notch signaling and the cell-communication network.

**Fig. 4:**
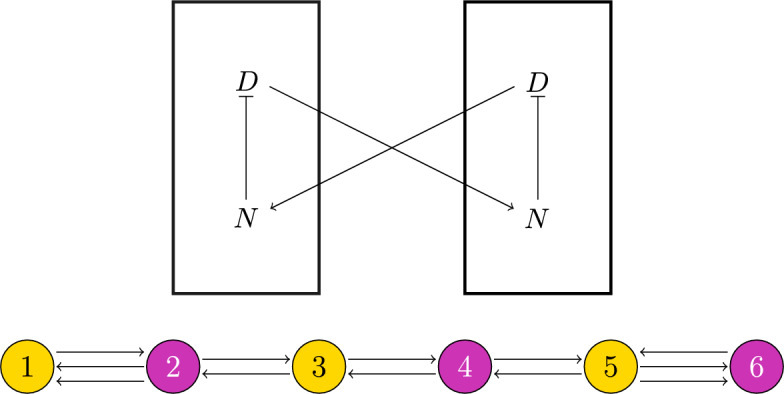
Top: A schematic diagram capturing the known qualitative features of Delta-Notch chemical signaling. Notch inhibits Delta within a cell, and Delta activates Notch in neighboring cells. Bottom: At one stage in development, the *C. elegans* vulva is a line of six cells. Under a mutation of the let-23 gene, each cell receives approximately the same amount of external signaling, so we can assume that their dynamics are the same; thus, the system can be represented with a regular network. The alternating color is the balanced coloring $\bowtie$ associated with the critical pattern space $P^{\mu _1}$ for the Notch pathway

#### Critical pattern space of Notch signaling is $P^{\mu _1}$

Following the model of Collier et al. (1996), the feedback between Delta and Notch in coupled cells can be represented as in Fig. 4 (top) where Notch inhibits Delta within a cell and Delta activates Notch in neighboring cells. It is natural to assume that *i* *activates* *j* (represented with $i \rightarrow j$) means that

$$\begin{aligned} \frac{\partial \dot{x}_j}{\partial x_i} > 0, \end{aligned}$$

and *i* *inhibits* *j* (represented with ) means that

$$\begin{aligned} \frac{\partial \dot{x}_j}{\partial x_i} < 0. \end{aligned}$$

Furthermore, we assume that Delta and Notch both decay over time, meaning that

$$\begin{aligned} \frac{\partial \dot{N}}{\partial N}&< 0 \\ \frac{\partial \dot{D}}{\partial D}&< 0. \end{aligned}$$

Writing the concentrations of the biochemical species as a vector (*D*, *N*), the internal and coupled dynamics of the Delta-Notch signaling pathway take the form

$$\begin{aligned}&\textbf{Q}=\begin{pmatrix} - & \quad - \\ 0 & \quad - \end{pmatrix} &  \textbf{R}=\begin{pmatrix} 0 & \quad 0 \\ + & \quad 0 \end{pmatrix} \end{aligned}$$

where $+$ denotes a positive term, and − denotes a negative term. Thus, we have $\det (\textbf{R})=0$; if $\textbf{A}$ has real eigenvalues, we can apply Theorem 10.

We also have that

$$\begin{aligned}&B=\text {tr}(\textbf{Q})\text {tr}(\textbf{R}) - \text {tr}(\textbf{QR}) > 0 \\&\text {tr}(\textbf{Q})<0=-|\nu \text {tr}(\textbf{R})| &  \text {(NDG condition, Theorem 11)}, \end{aligned}$$

so from Table 1, the critical pattern space is $P^{\mu _1}$. This is a natural result since nondegenerate synchrony-breaking bifurcations have critical pattern space $P^{\mu _1}$ (Corollary 11.1).

#### Preferred pattern in mutated *C. elegans* vulval precursor cells (VPCs)

The *C. elegans* vulval precursor cells (VPCs) form a pattern that is mediated by Notch signaling. With a mutation of let-23, all VPCs receive approximately equal external signals (Aroian and Sternberg 1991), so the system can be represented with the regular network in Fig. 4 (bottom). The adjacency matrix is

$$\begin{aligned} \textbf{A} = \begin{pmatrix} 0& \quad 2& \quad 0& \quad 0& \quad 0& \quad 0\\ 1& \quad 0& \quad 1& \quad 0& \quad 0& \quad 0\\ 0& \quad 1& \quad 0& \quad 1& \quad 0& \quad 0\\ 0& \quad 0& \quad 1& \quad 0& \quad 1& \quad 0\\ 0& \quad 0& \quad 0& \quad 1& \quad 0& \quad 1\\ 0& \quad 0& \quad 0& \quad 0& \quad 2& \quad 0 \end{pmatrix}. \end{aligned}$$

Using Matlab, we find that the eigenvalues are real, and the smallest eigenvalue and its corresponding eigenvector are

$$\begin{aligned} \mu _1&=-2&\textbf{v}_1&= (1,-1,1,-1,1,-1), \end{aligned}$$

implying that the critical pattern space $P^{\mu _1} = \text {span}_{\mathbb {R}}\{\textbf{v}_1\}$. $\mu _1$ has geometric multiplicity 1, so the critical eigenspace is 1-dimensional (Table 1), and the critical pattern space $P^{\mu _1}$ corresponds to the balanced coloring $\bowtie$ shown in Fig. 4 (bottom), so Theorem 3 implies that there is a bifurcating branch of solutions with the pattern $\bowtie$. Furthermore, Proposition 5 suggests that this will be the only stable branch near the bifurcation, so we expect the *C. elegans* vulva, with a mutation of let-23, to exhibit an alternating pattern of cell fates – and this is exactly what is observed experimentally.

#### Preferred patterns in square arrays of cells developing according to Notch signaling

**Fig. 5:**
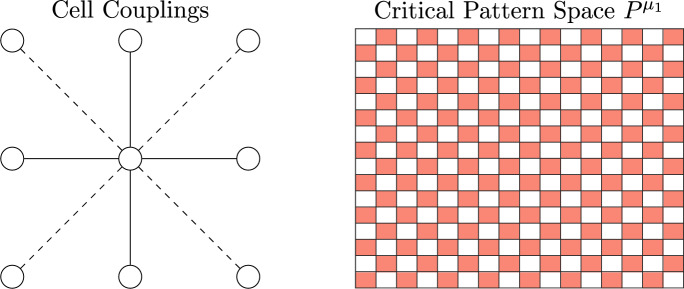
We consider a $16 \times 16$ array of cells that develops according to the Notch pathway with nearest and next nearest neighbor couplings of cells, as shown on the left. Our theory predicts that the cells will form the checkerboard pattern on the right, which matches our simulations

**Fig. 6:**
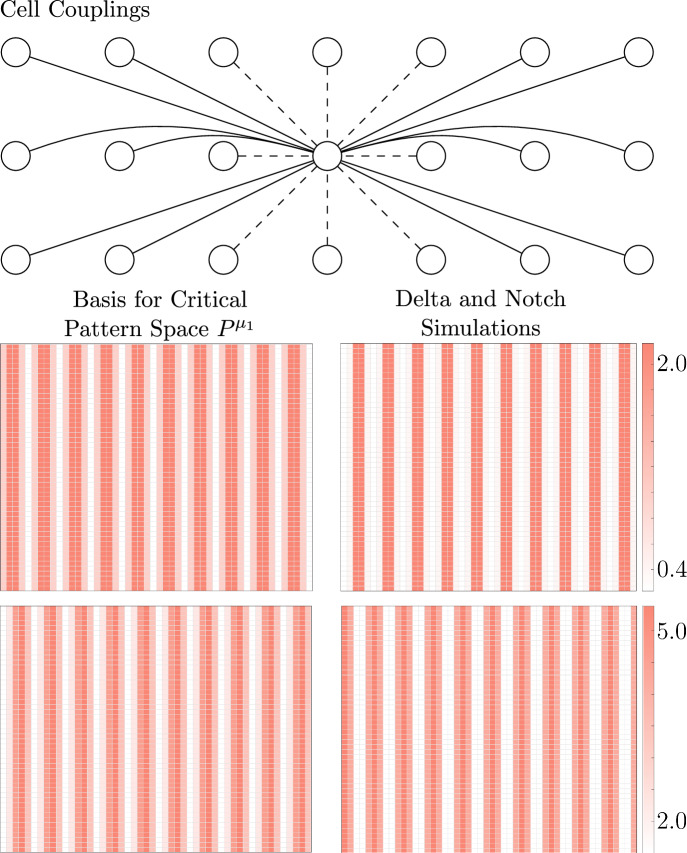
Now we consider a $50\times 50$ array of cells that can communicate over long ranges as shown on the top. The critical pattern space is the 2-dimensional space spanned by the vectors on the left; however, we observe that $\text {dim}(G^{\mu }\cap \Delta _{\bowtie })=1$ for the critical generalized eigenspace $G^{\mu }$ and the pattern $\bowtie$ given by the first basis vector. Therefore, Theorem 3 implies that generically there is a unique branch of bifurcating solutions with synchrony pattern $\bowtie$, which matches our simulations of Delta and Notch (top right and bottom right, respectively)

We will compute the critical pattern space of two square arrays of cells that develop according to Notch signaling (Figs 5, 6), showing that cells can change their communication to form a different pattern despite using the same signaling mechanism.

We have shown that the critical pattern space for the Notch pathway is $P^{\mu _1}$, so we must compute $P^{\mu _1}$ to determine the preferred pattern of the tissue. $P^{\mu _1}$ depends on the cell-communication network. In Fig. 5, we consider a $16\times 16$ array of cells, where each cell is coupled to its neighbors as depicted on the left (for clarity, we omit arrows pointing towards the central cell), and there are periodic boundary conditions. Dotted lines represent a connection strength of 1, and solid lines represent a connection strength of 3. Since there are periodic boundary conditions, the adjacency matrix $\textbf{A}$ will be real and symmetric, so $\textbf{A}$ will have real eigenvalues.

Using Matlab, we find that the smallest eigenvalue $\mu _1$ has algebraic multiplicity one. Its corresponding eigenvector $v_1$ is contained in the balanced coloring given by the checkerboard pattern $\bowtie$ in Fig. 5 (right), so $\text {dim}(\text {span}_{\mathbb {R}}\{v_1\} \cap \Delta _{\bowtie })=\text {dim}(\text {span}_{\mathbb {R}}\{v_1\})=1$. By Theorem 3, generically there is a unique branch of bifurcating solutions with the checkerboard pattern, which matches our simulations.

In Fig. 6, we consider a $50 \times 50$ array of cells with couplings shown in the top panel and periodic boundary conditions. Using Matlab, the minimal eigenvalue $\mu _1$ has algebraic multiplicity 2, and the critical pattern space is the 2-dimensional space spanned by the vectors depicted on the left of Fig. 6. We observe, however, that $\text {dim}(G^{\mu } \cap \Delta _{\bowtie })=1$ where $G^{\mu }$ is the critical generalized eigenspace, and $\bowtie$ is the coloring given by the top eigenvector in the basis (Fig. 6). Thus, we expect to see the pattern $\bowtie$, which our simulations validate, as shown on the right of Fig. 6.

### Inferring biochemical interactions from observed patterns

Finally, we show how the theory can be used to infer properties of the chemical signaling pathway from an observed pattern.

**Fig. 7:**
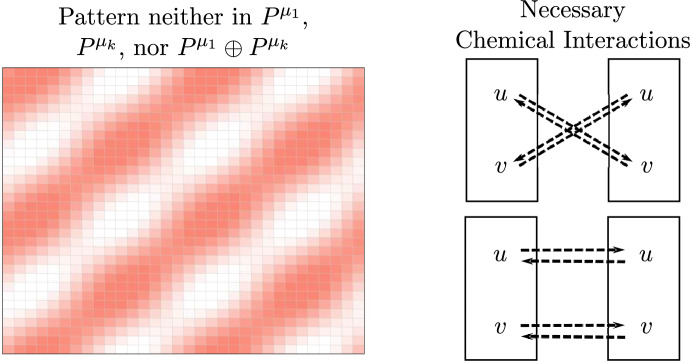
Assuming that the cells are coupled as in Fig. 5 (left), the given pattern is neither $P^{\mu _1}$, $P^{\mu _k}$, nor $P^{\mu _1} \oplus P^{\mu _k}$. Barring the degenerate case that the critical pattern space is $\mathbb {R}^n$, by Theorem 13 we must have that $\det (\textbf{R}) > 0$, so the developed pattern must be due to a chemical signaling network with either of the connections on the right

For a $16 \times 16$ array of cells communicating as in Fig. 5 (left), if we observe the striped pattern in Fig. 7 (left), then the critical pattern is neither $P^{\mu _1}$, $P^{\mu _k}$, nor $P^{\mu _1} \oplus P^{\mu _k}$. Degeneracies are unlikely to occur in biological sytems, and if we rule out the extremely degenerate case that the critical pattern space is $\mathbb {R}^n$, then we must have $\det (\textbf{R}) > 0$ by the contrapositive of Theorem 13. Thus, the developed pattern must be due to a chemical signaling network with the connections given on the right of Fig. 7 (top or bottom).

**Fig. 8:**
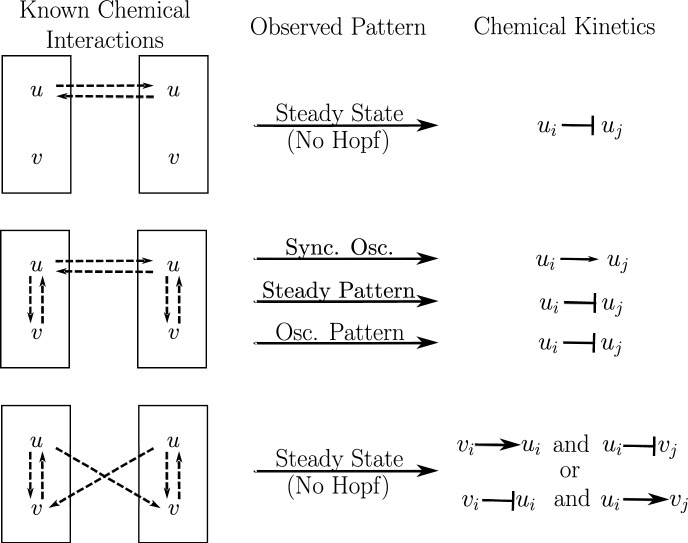
In section 3.3, we infer qualitative features of chemical kinetics given partial information about the chemical signaling network and the type of pattern that forms in a tissue. The analysis is summarized above. “Sync. osc.” is short for “synchronous oscillations” and “osc. pattern” is short for “oscillating pattern”

Now, suppose there is some regular array of uniform cells. If we have partial information about chemical signaling in a tissue, we can use the observed tissue pattern to obtain additional information about chemical signaling by using the following steps.

1. Use the partial information about chemical interactions to create matrices representing the internal dynamics $\textbf{Q}$ and coupled dynamics $\textbf{R}$ with variables as entries.
2. Use Theorems 7 and 8 to determine the signs of several variables in $\textbf{Q}$ and $\textbf{R}$.
3. Check $\det (\textbf{R})=0$. Then reference the observed pattern against Table 1 to determine necessary conditions on *B* and $\textbf{R}$.
4. Use newfound information about *B* and $\text {tr}(\textbf{R})$ to determine the possible signs of additional variables in $\textbf{Q}$ and $\textbf{R}$.
5. Lastly, convert the information about the sign of entries in $\textbf{Q}$ and $\textbf{R}$ to information about activation and inhibition of biochemical species.

For the first example, consider when one chemical *u* in a cell influences the same chemical in a neighboring cell, and suppose that the chemical is independent of other chemical species. Then the node space is 1-dimensional, and we can represent the internal and coupled dynamics as in Fig. 8 (top). This tells us that

$$\begin{aligned} \textbf{Q}&= \frac{\partial \dot{u}_i}{\partial u_i} \\ \textbf{R}&= \frac{\partial \dot{u}_i}{\partial u_j}. \end{aligned}$$

For the uniform or synchronous state to be stable, we must have that $\textbf{Q} < 0$ (Proposition 7), implying that *u* decays. Assume that *u* decays at the same rate for all time. For a pattern to form, there must be a synchrony-breaking bifurcation. By Theorem 9 a nondegenerate synchrony-breaking bifurcation occurs if and only if $\textbf{R}<0$, meaning that *u* in one cell must inhibit *u* in its neighboring cells, and the inhibition must increase for a synchrony-breaking bifurcation to occur.

Now, suppose we know that the concentration of *u* is affected by the concentration of another chemical *v* within the cell (Fig. 8, middle). Then writing the chemical species as a vector (*u*, *v*),

$$\begin{aligned} \textbf{Q}&= \begin{pmatrix} a & \quad b \\ c & \quad d \end{pmatrix} \\ \textbf{R}&= \begin{pmatrix} \kappa & \quad 0 \\ 0 & \quad 0 \end{pmatrix} \end{aligned}$$

for some real numbers $a,b,c,d,\kappa$.

If the cells are initially uniform, then from Proposition 8,

$$\begin{aligned} \text {tr}(\textbf{Q}) = a+d < 0. \end{aligned}$$

This is satisfied if both chemical species decay – as is standard. Furthermore, notice that

$$\begin{aligned} \det (\textbf{R})&= 0, \end{aligned}$$

so we can apply Theorem 10. If the cells oscillate in sync, then we may assume that there was a nondegenerate synchrony-preserving Hopf bifurcation, implying that $\text {tr}(\textbf{R})=\kappa > 0$ (Table 1, row 4), so $u_i \rightarrow u_j$. If they oscillate in a pattern, then we may assume there was a nondegenerate synchrony-breaking Hopf bifurcation, implying that $\text {tr}(\textbf{R})=\kappa < 0$ (Table 1, row 2) and thus . If we see that a steady state pattern has formed among the cells, then we can infer that a nondegenerate synchrony-breaking steady state bifurcation occurred (Table 1, row 1) meaning that

$$\begin{aligned} B&= \text {tr}(\textbf{Q})\text {tr}(\textbf{R}) - \text {tr}(\textbf{QR}) \\&= (a+d)\kappa - a\kappa \\&= d\kappa >0. \end{aligned}$$

Assuming that *v* decays, $d<0$, implying that  and the strength of decay of *v* or the strength of inhibition must increase for a pattern to form.

Lastly, assume that *u* in cell *i* influences *v* in neighboring cells *j* as shown by the general diagram in Fig. 8 (bottom). Writing the chemical species as a vector (*u*, *v*), we have that the internal and coupled dynamics are given by

$$\begin{aligned} \textbf{Q}&= \begin{pmatrix} a & \quad b \\ c & \quad d \end{pmatrix} \\ \textbf{R}&= \begin{pmatrix} 0 & \quad 0 \\ \kappa & \quad 0 \end{pmatrix} \end{aligned}$$

for real numbers $a,b,c,d,\kappa$.

If the cells are initially uniform, then from Proposition 8,

$$\begin{aligned} \text {tr}(\textbf{Q}) = a+d < 0. \end{aligned}$$

Again, this is satisfied if both chemical species decay. Furthermore, notice that

$$\begin{aligned} \det (\textbf{R})&= 0 \\ \text {tr}(\textbf{Q})&< -|\nu \text {tr}(\textbf{R})| = 0 &  \text {(NDG condition, Theorem 11)}, \end{aligned}$$

so applying Theorem 10, we are in the first or third row of Table 1. This means there can only be a steady state bifurcation (i.e. no oscillatory behavior). And

$$\begin{aligned} B = \text {tr}(\textbf{Q})\text {tr}(\textbf{R}) - \text {tr}(\textbf{QR})=-b\kappa . \end{aligned}$$

If $\text {sgn}(b)=\text {sgn}(\kappa )$, then synchrony cannot be broken since $B<0$ (see Table 1). If we observe a pattern, however, then we expect that $\text {sgn}(b)\ne \text {sgn}(\kappa )$, meaning that we either have

1. $$v_i \rightarrow u_i$$ and
2. or  and $$u_i \rightarrow v_j$$.

## Discussion

This framework provides powerful tools for molecular biologists who are interested in uncovering the mechanisms of pattern formation. It provides a systematic approach to identifying the molecular causes of pattern failures, which can help guide protein knockdown experiments in model organisms. Additionally, our theory can help uncover unknown molecular interactions in chemical signaling networks through the analysis of patterns that emerge in tissues (Fig. 8).

This framework was developed by recognizing that a developing tissue can be modeled as as a system of ODEs on a regular network. When the linearization has null eigenvalues, a pattern can form through a bifurcation (Theorem 3). Using the network framework, we can determine the eigenvalues by examining smaller matrices $\textbf{Q}+\mu _i \textbf{R}$, where $\mu _i$ represents an eigenvalue of the adjacency matrix $\textbf{A}$, while $\textbf{Q}$ and $\textbf{R}$ represent the linearized internal and coupled dynamics, respectively (Proposition 2). Thus, the pattern is determined by both the global cell-communication structure ($\mu _i$) and the local cell-level dynamics ($\textbf{Q}$ and $\textbf{R}$).

We use our assumptions about a developing tissue to gain information about the pattern and chemical kinetics. Since the synchronous state is initially stable, all eigenvalues are negative, which provides crucial information about the chemical kinetics described by $\textbf{Q}$ and $\textbf{R}$ (Propositions 7, 8). Then for a pattern to form, $\textbf{Q}+\mu _i \textbf{R}$ must have a null eigenvalue for some $\mu _i$. We found that in nondegenerate systems with either one or two biochemicals and one coupling between cells, the resulting steady state pattern always corresponds to the smallest eigenvalue of $\textbf{A}$ (denoted $\mu _1$) and is given by the critical pattern space $P^{\mu _1}$ (Definition 10). The formation of the pattern, however, requires specific conditions on $\textbf{Q}$ and $\textbf{R}$ (Theorems 9, 10).

Altogether, our theory suggests that (1) if the chemical kinetics $\textbf{Q}$ and $\textbf{R}$ satisfy certain conditions, then a pattern $P^{\mu _1}$ will form that is dictated by the cell-communication structure $\textbf{A}$ (Section 3.2); and (2) if a pattern $P^{\mu _i}$ forms in an array with known communication structure $\textbf{A}$, then the chemical kinetics $\textbf{Q}$ and $\textbf{R}$ must satisfy certain properties (Section 3.3).

Our framework also extends beyond basic research with several potential applications. In tissue engineering, our findings suggest that controlling the range of cell-communication can guide pattern formation (Section 3.2.3). Alternatively, maintaining the same communication structure while adjusting chemical signaling can alter the tissue pattern (Figure 7). In medicine, these insights have important implications for understanding disease mechanisms, as disruptions in pattern formation are associated with congenital malformations. We provide a theoretical framework for understanding the molecular changes underlying these failures. In particular, our theory illustrates that multiple molecular factors can lead to the same pattern breakdown, which may explain why drugs succeed in treating a disease in some individuals while failing in others, and suggests novel drug targets.

Although our framework relies on simplified assumptions about biological systems, future research will refine and expand its applicability. A key next step will be extending our analysis from two-dimensional signaling networks to more complex, realistic biochemical systems. This expansion will improve our ability to identify the molecular causes of pattern formation. Additionally, we plan to validate our theoretical predictions through simulations that incorporate spatial features including morphogen gradients, parameter noise, and network perturbations, enabling us to apply our theory to a broader range of biological contexts.

## Data Availability

The code referenced in this paper is available on Github at https://github.com/ldobrien1234/cell-pattern-validation. It contains the Matlab code used to find the critical pattern space in a square array of cells and simulations that verify our theoretical predictions as shown in Figures 5,6. All figures were created using Inkscape and TikZ.
